# Reference Gene Selection for RT-qPCR Analysis of Flower Development in *Chrysanthemum morifolium* and *Chrysanthemum lavandulifolium*

**DOI:** 10.3389/fpls.2016.00287

**Published:** 2016-03-11

**Authors:** Shuai Qi, Liwen Yang, Xiaohui Wen, Yan Hong, Xuebin Song, Mengmeng Zhang, Silan Dai

**Affiliations:** College of Landscape Architecture, Beijing Forestry UniversityBeijing, China

**Keywords:** *Chrysanthemum morifolium*, *Chrysanthemum lavandulifolium*, reference genes, qRT-PCR, flower development

## Abstract

Quantitative real-time PCR (qPCR) is a popular and powerful tool used to understand the molecular mechanisms of flower development. However, the accuracy of this approach depends on the stability of reference genes. The capitulum of chrysanthemums is very special, which is consisting of ray florets and disc florets. There are obvious differences between the two types of florets in symmetry, gender, histological structure, and function. Furthermore, the ray florets have various shapes. The objective of present study was to identify the stable reference genes in *Chrysanthemum morifolium* and *Chrysanthemum lavandulifolium* during the process of flower development. In this study, nine candidate reference genes were selected and evaluated for their expression stability acrosssamples during the process of flower development, and their stability was validated by four different algorithms (Bestkeeper, NormFinder, GeNorm, and Ref-finder). *SAND* (SAND family protein) was found to be the most stably expressed gene in all samples or different tissues during the process of *C. lavandulifolium* development. Both *SAND* and *PGK* (phosphoglycerate kinase) performed most stable in Chinese large-flowered chrysanthemum cultivars, and *PGK* was the best in potted chrysanthemums. There were differences in best reference genes among varieties as the genetic background of them were complex. These studies provide guidance for selecting reference genes for analyzing the expression pattern of floral development genes in chrysanthemums.

## Introduction

Asteraceae is one of the largest families of flowering plants (Tähtiharju et al., 2012). Members of this family have typical capitulum, which is consists of ray florets and disc florets. Ray florets are highly specialized in pollinator attraction and disc florets assume the reproductive function. The combination of two types florets improve the chances of reproductive success and are more evolved than a single flower (Lane, 1994; Funk et al., 2009). In addition, there are obvious differences between the two types of florets in symmetry, gender, histological structure, and function. Chrysanthemum (*Chrysanthemum* × *morifolium* Ramat.), one of the 10 most well-known Chinese traditional flowers, is composed of the most abundant and various cultivars among the horticultural plants around the world (Dai et al., 2005). The petal types of chrysanthemums were classified into five groups containing flat, tubular, spoon, abnormal, and anemone (Chen, 2001). The diversity of the flower shape is determined by the numbers of the two types of florets and the variation of petal types. The regulatory mechanism controlling chrysanthemum flower shape is still not clear yet. Hence, analysis the expression patterns of key genes involved in the flower development of chrysanthemums will help us to understand the mechanism of flower development and promote molecular breeding in chrysanthemum flower shape. Chrysanthemums are classified as a hybrid cultigen complex after a long period of inter-specific hybridization and artificial selection (Chen, 1957, 1984; Dai et al., 2002). We then selected *Chrysanthemum lavandulifolium* (Fisch. ex Trautv.) Makino, one of the original species of *C. morifolium*, which has simple genetic background than most composite plants (Zhang and Dai, 2009), as a model plant for molecular biological research on chrysanthemums.

The conserved function of genes in flower development has provided some clues. Quantitative real-time RT-PCR (qPCR) is a reliable technique for gene expression analysis that is used in many research fields because of its sensitivity, accuracy, specificity, and high throughput (VanGuilder et al., 2008; Bustin et al., 2009; Artico et al., 2010). It has been widely used in researches on flower development in higher plants (Artico et al., 2010; Mallona et al., 2010). It is necessary to select reliable reference genes to normalize RT-qPCR analyses. An improper reference gene can lead to misinterpretation of expression data and thus generate incorrect results (Dheda et al., 2005). Many studies have been conducted to evaluate the stability of potential reference genes in plants, such as *Arabidopsis thaliana* (Czechowski et al., 2005; Remans et al., 2008; Dekkers et al., 2012), rice (Kim et al., 2003; Jain et al., 2006), wheat (Paolacci et al., 2009), tobacco (Schmidt and Delaney, 2010), and soybean (Jian et al., 2008; Libault et al., 2008; Hu et al., 2009; Kulcheski et al., 2010). A few studies have been reported in composite plants. *MTP* (Metalloprotease), *SKIP16* (SKP1/ASK-interacting protein 16), and *PGK* were the most stable genes in different tissues at various developmental stages and in leaves, with varied photoperiodic treatments in *C. lavandulifolium* (Fu et al., 2013). *F-box* (*F*-box protein) and *PP2A* (protein phosphatase 2A) were the most stable genes in three flower color lines at five floral developmental stages and at two light conditions in *C*. × *morifolium* “Reagan” (Hong and Dai, 2015). *EF-1a* (elongation factor 1α) and *PGK* were the best combination for four taxa including diploid level and polyploid level in *C. nankingense* and *C. zawadskii* (Wang et al., 2015). *TIP41* (TIP41 like protein) was shown to be the most stable gene in *Cichorium intybus* (Delporte et al., 2015). In chrysanthemum cultivar “Zhongshanzigui,” *EF1a* performed well for aphid infested plants but poorly for waterlogged ones. Furthermore, *PP2Acs* performed best during heat and waterlogging stress, but was the worst during aphid infestation (Gu et al., 2011). Nevertheless, there was limited information available concerning reference genes for flower shape in chrysanthemums.

Flower development in higher plants has fascinated researchers for a long time. It is one of the 125 most compelling puzzles and questions facing scientists today (Miller, 2005). We aimed to identify potential reference genes suitable for transcript normalization in different florets and ray florets of different petal types during chrysanthemum flower development and then validate their stability. These reference genes will enable more accurate and reliable RT-qPCR normalization for gene expression studies in chrysanthemum flower development.

## Materials and methods

### Plant materials

Organs of different developmental stages were collected from *C. lavandulifolium* (set1), four Chinese large-flowered chrysanthemum cultivars (set2), and three chrysanthemum hybrids of potted chrysanthemum (set3).

#### C. lavandulifolium

Seeds of *C. lavandulifolium* were collected from the campus of Peking University. They were germinated in plugs filled with vermiculite medium for 30 days and transferred to 9 × 9 cm flowerpots with turf and vermiculite (1/1 v/v) media under LD conditions (16 h light/8 h dark). At the end of their juvenile period, the seedlings with 14 leaves were placed in short-day (SD) conditions (12 h light/12 h dark). The room temperature was kept at 22 ± 1°C and relative air humidity of 60%.

**Figure 1:**
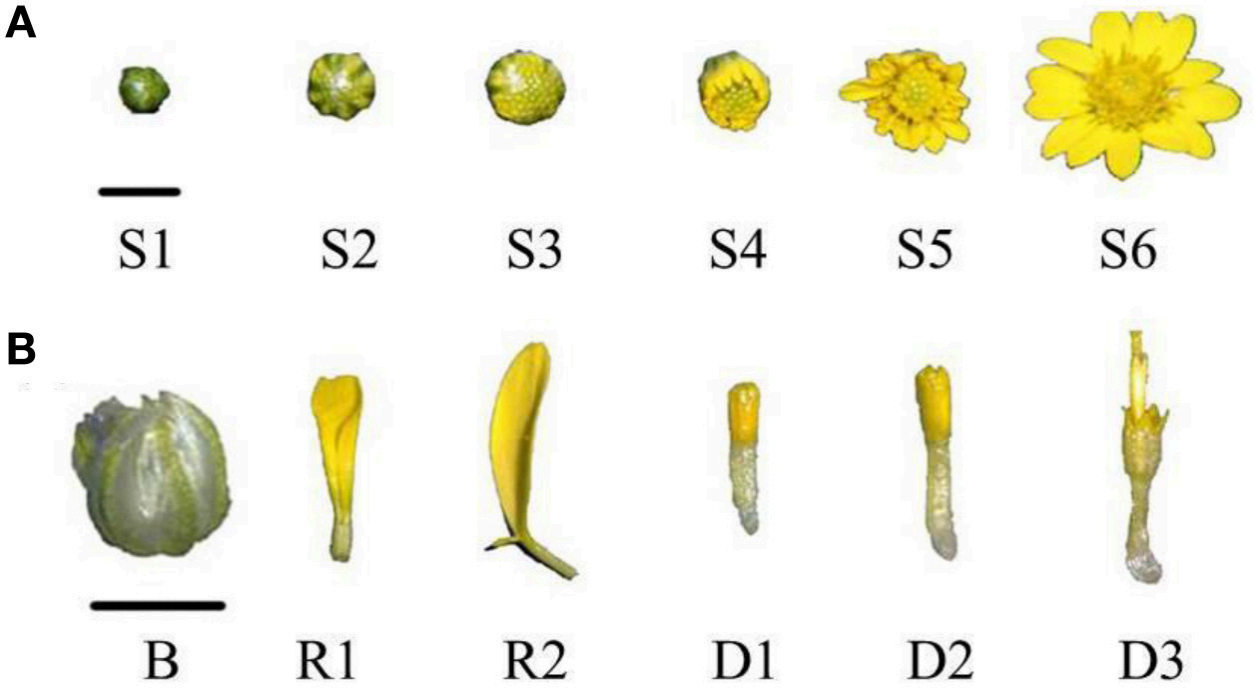
**The definitions of the stages of ***C. lavandulifolium*** flower development. (A)** Six stages of *C. lavandulifolium* capitulum development, **(B)** stages of *C. lavandulifolium* flower organ development. Bar = 0.5 cm.

The development of the flower was divided into six different developmental stages (Figure 1, Table 1). The development of the ray floret was divided into two stages, and the development of the disc floret was divided into three stages as their development were not synchronized (Figure 1, Table 1).

**Table 1:** **The phenotype of ***C. lavandulifolium*** flower developmental stages**.

**Developmental stage**	**Phenotype**
S1	The diameter of flower bud is <3 mm and bracts are not opened
S2	The diameter of flower bud is more than 3 mm and bracts are not opened
S3	The flower bud opens while ray florets are not visible yet
S4	The flower bud opens and the tips of florets are visible
S5	The outer florets elongate and are obliquely oriented
S6	The outer florets are horizontally oriented
B	Bracts outer of the capitulum
R1	Ray florets of S4 stage, out of the bracts
R2	Ray florets of S6 stage, which have reached the maximum length
D1	Green disc florets before S4 stage, which are immature
D2	Disc florets with mature pollen
D3	Disc florets with pollen gone out

#### C. morifolium

**Figure 2:**
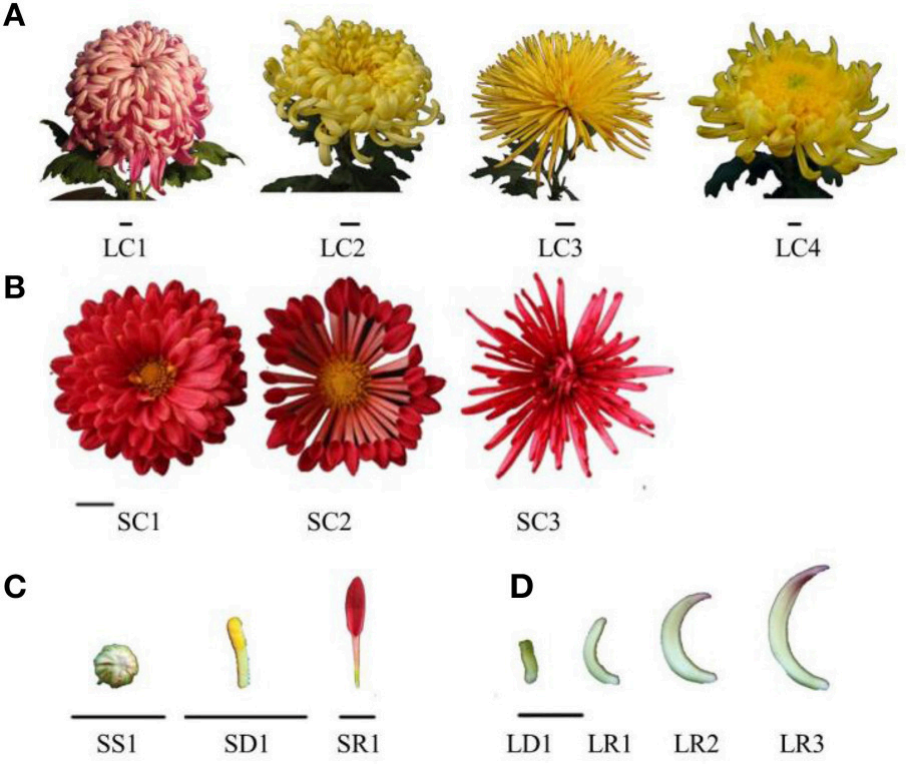
**Inflorescence of four chrysanthemum cultivars and three chrysanthemum hybrids. (A)** Chinese large-flowered chrysanthemum cultivars of four-petal type; **(B)** potted chrysanthemum of three petal types; **(C,D)** sample of chrysanthemum flower. Bar = 1 cm.

We selected four chrysanthemum varieties (Chinese large-flowered chrysanthemum cultivars) and three chrysanthemum hybrids (potted chrysanthemum), which covers four typical petal types (Figure 2, Tables 2, 3). They can be used to study gene expression of two florets and the different petal types. These materials were obtained from the Chrysanthemum Nursery of Beijing Forestry University, Beijing, China. Robust cutting slips were cut from mother plants and stuck in plugs (turf: vermiculite = 3:1, v/v) in April. Seedlings with strong roots were transferred to pots after 25 days. Water, fertilizer, insects, and disease control were supplied during this period. The whole process of growth was in natural conditions and chrysanthemums blossomed in October.

**Table 2:** **Parameters of four chrysanthemum cultivars and three chrysanthemum hybrids**.

**No**.	**Name**	**Petal type**	**Petal color**	**Class**	**Set**
LFC1	“sayuhuaqing”	Flat	Red	Chinese large-flowered chrysanthemum cultivars	set2
LFC2	“dapengzhanchi”	Spoon	Yellow		
LFC3	“huangxiangli”	Tubular	Yellow		
LFC4	“kuihuatuogui”	Anemone	Yellow		
PC1	–	Flat	Red	Potted Chrysanthemum	set3
PC2	–	Spoon	Red		
PC3	–	Tubular	Red		

**Table 3:** **The phenotype of the samples of chrysanthemum flower**.

**Set**	**Stage**	**Phenotype**
Set2	LR1	Ray florets of stage S2
	LR2	Ray florets of stage S3
	LR3	Ray florets of stage S4
	LD1	Disc florets of stage D1
Set3	SS1	Capitulum of stage S1
	SR1	Ray florets of stage R1
	*SD*1	Disc florets of stage D1

### RNA isolation and cDNA synthesis

Total RNA was extracted from all samples using Quick RNA Isolation Kit (HUAYUEYANG, China). All RNA samples were adjusted to the same concentration after measuring the RNA concentration on a NanoDrop ND-1000 spectrophotometer (NanoDrop Technologies). The integrity of the RNA was further verified by 1% (w/v) agarose gel electrophoresis and ethidium bromide staining. All samples were pretreated with an RNase-free DNase I (Promega, USA) at 37°C for 30 min to eliminate any DNA contamination. The first strand cDNA was synthesized based on 1 μg of total RNA using the M-MLV reverse transcription system (Promega, USA) according to the manufacturer's protocol.

### PCR primer design and test of amplification efficiency

A synthetic comparison of the reference genes among different composite plants was taken and nine of them were selected as our candidate reference genes (*Actin, EF-1*α, *PP2A, SAND, MTP, PGK, TUB, UBQ*) as they performed well in the composite plants in previous researches (Gu et al., 2011; Fu et al., 2013; Jin et al., 2013; Hong and Dai, 2015; Wang et al., 2015; Table 4). Five primers have been reported in *C. lavandulifolium* or *C. morifolium* and the other four primers were designed using Primer5 software with melting temperatures (Tm) of 55–65°C, primer lengths of 19–26 bp and amplicon lengths of 197–297 bp (Table 4). The performance of the primers was tested by RT-PCR and the products of the primer amplicons was verified by 2% (w/v) gel electrophoretic analysis.

**Table 4:** **Reference gene primer sequences and amplicon characteristics**.

**Gene symbol**	**Gene description**	**Forward primer sequences (5′–3′)**	**Reverse primer sequences (5′–3′)**	**Amplicon Length (bp)**	**Tm (°C)**	**PCR efficiency (%)**	**Regression Coefficient (R2)**	**Mean of Cq value**	**References**
*Actin*	Actin related protein 2	TAAGAACGATAAGTGCCCACATAG	TTTTAGACATCAGCCATAACAGAGT	201	62.1	122.7	0.992	27.76 ± 1.32	Fu et al., 2013
*EF-1α*	Elongation factor 1 alpha	TGCCGTTGGGGTGAGTATT	CCTTCTTTTCCTCCTCGGTCT	179	57	109.3	0.995	22.97 ± 1.35	
*PP2A*	Protein phosphatase 2A	CGTGGGTCCTCAGAATCAAA	TGTCAGCCATCTGTAAAATG	297	55.1	93.6	0.994	25.24 ± 1.15	Hong and Dai, 2015
*SAND*	SAND family protein	CGTTGCTCACTACGAGTTCAC	GCAGATGGGTCAACAGGTAA	178	62.1	124.0	0.995	26.04 ± 1.08	Fu et al., 2013
*MTP*	Protein degradation	GGTGTTATGATTGGTGCTGCT	ATCTATCTCTCGTGGGGTGCT	184	57.2	131.6	0.995	25.11 ± 1.29	Fu et al., 2013
*PGK*	Phosphoglycerate kinase	ATACCTAATGGACGAAGAGA	TTATTGTCGTCTTTACTACCA	248	64.1	113.5	0.999	27.54 ± 1.19	Fu et al., 2013
*TUB*	beta tubulin	ATCAACTACCAGCCACCAAC	GAACTCACCTTCCTCCATACC	211	58	107.9	0.990	22.09 ± 1.28	
*UBQ*	ubiquitin extension protein	GAACCATCCACGAACAATACAAGAGC	CAGGCTAAGAGGAGGGATGC	283	56.2	110.7	0.999	19.29 ± 1.20	
*F-box*	F-box protein	CTGCTTTATGTATCGGAGGGA	AACGAAAACGAGAGGGGCT	246	58.3	95.9	0.995	23.15 ± 1.30	

### Quantitative RT-PCR analysis

PCR reactions were performed using a Mini Opticon Real-time PCR System (Bio-Rad, USA) based on SYBR Premix Ex Taq (TaKaRa, Japan). Reactions with 20 μl total volume contained 2 μl of template (~50 ng), 0.4 μl of each amplification primer (10 μM), 10 μl of 2X SYBR Premix Ex Taq, and 7.2 μl of ddH_2_O. The amplification program was at 95°C for 30 s, and then 40 cycles of 95°C for 5 s, an optimized annealing temperature for 30 s and 72°C for 30 s. Melting curves were recorded after cycle 40 by heating from 65 to 95°C, increasing the temperature stepwise by 0.5°C every 5 s. Each experiment was repeated three times with three biological replicates. No template controls were performed with double distilled water as template.

### Data analysis

Four different VBA (Visual Basic Applet) applets were used to rank the stability of nine reference genes using the above Cq or raw Ct-values in all experimental sets in this study: GeNorm (Vandesompele et al., 2002), NormFinder (Brunner et al., 2004), Bestkeeper (Pfaffl et al., 2004), and Ref-finder (http://www.leonxie.com/referencegene.php). The GeNorm software calculates the gene expression stability (*M*) based on the average pairwise variation (*V*) among all other reference genes. The expression stability of reference genes was ranked through stepwise exclusion of the least stable gene and recalculation of the average *M*-values until only two genes were left. A low *M*-value defined to represent stable expression of the individual gene. The NormFinder program uses an ANOVA-based model to estimate intra- and inter-group variation and ranked the reference genes according to their stability in a given sample set. Bestkeeper ranks the reference genes based on the calculation of coefficient of variance (*CV*) and the standard deviation (*SD*) of the Cq-values (Pfaffl et al., 2004). The reference genes are identified as the most stable when they exhibit the lowest *CV* and *SD* [*CV* ± *SD*]. Ref-finder (http://www.leonxie.com/referencegene.php) was used to confirm the reliability of the calculation. Ref-finder is a web-based tool that integrates the current major computational programs, including GeNorm, NormFinder, Bestkeeper, and the comparative ΔCT method, to compare and rank the stability of candidate reference genes.

### Assessment of normalization

Flower color of seven chrysanthemum cultivars and *C. lavandulifolium* are typically red or yellow, which means the main pigments are anthocyanin or carotenoid. In order to validate the reference genes identified in this study, we evaluated the expression of *DmDFR* (dihydroflavonol 4-reductase) and *LCYE* (lycopene e-cyclase) genes (**Table 9**). *DmDFR* and *LCYE* play an important role in the anthocyanin biosynthesis pathway and carotenoid biosynthesis pathway, respectively. The homologs of *LCYE* and *DFR* were previously characterized by RT-PCR.

## Results

### Performance of the primers for each reference gene

Melting curve analysis was performed by qPCR after 40 cycles of amplification (Figure S2). The presence of a single peak indicated that the expected amplicons were amplified (Figure S1). The correlation coefficients (*R*^2^) ranged in value between 0.992 and 1.0000, and PCR amplification efficiencies between 93.6 and 131.6% were obtained from the standard curves generated using a 10-fold serial dilution of cDNA (Figure S3). No template controls showed no peak and this proved there were no primer dimers.

The expression levels of the nine candidate reference genes presented as Cq-values are shown in Table 4. The mean values of the reference genes were between 19.29 and 27.76, which represented the different expression levels. *UBQ* showed the highest expression level in all samples and the lowest Cq-value (19.29). *Actin* presented the lowest expression level and the highest Cq-value (27.76)(Table S1).

### The stability of reference genes

To achieve a more accurate expression analysis, the 36 samples were divided into three experimental sets and analyzed individually. Set1 consists of 12 samples from *C. lavandulifolium*. Set2 was composed of 16 samples from Chinese large-flowered chrysanthemum cultivars. Set3 was composed of eight samples from potted chrysanthemum. In addition, we analyzed the overall stability among the three sets and every variety.

### GeNorm

**Figure 3:**
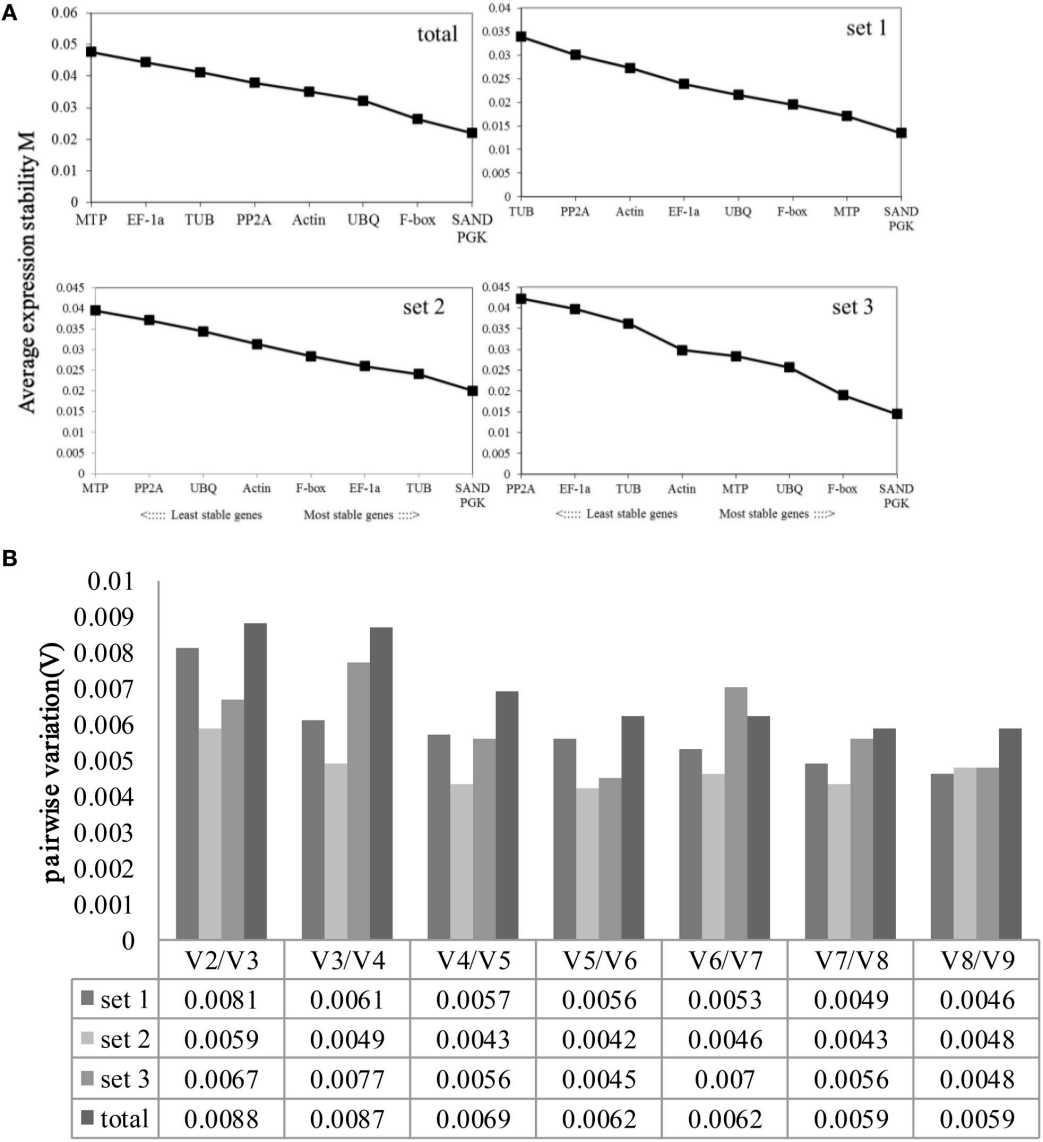
**Average expression stability values (***M***) and pairwise variation (***V***) of the candidate reference genes were calculated using GeNorm. (A)** Average expression stability values (*M*) following stepwise exclusion of the least stable gene across all treatment groups. The least stable genes are on the left, while the most stable genes are on the right. **(B)**. The optimal number of reference genes required for effective normalization. The pairwise variation (V_n_/V_n__+1_) was analyzed between the normalization factors NF_*n*_ and NF_*n*__+1_ using GeNorm.

The GeNorm program was used to rank the reference genes' expression stability by calculating the average expression stability (*M*). Stably expressed genes had values below 1.5 (Vandesompele et al., 2002). The ranking order according to the *M*-value is depicted in Figure 3.

All of the genes showed high expression stability, with *M*-values of <0.05. *SAND* and *PGK* were the most stably expressed genes, with an *M*-value of 0.022; *MTP* and *EF-1a* were the least stable genes in all samples (Figure 3). In set1, *SAND* and *PGK* performed well, with an *M*-value of 0.02; *MTP* and *PP2A* had high *M*-values. *SAND* and *PGK* were the most stable genes, with an *M*-value of 0.001; *TUB* and *PP2A* were the least stable genes in set2. In set3, *SAND* and *PGK* were the most highly ranked, with an *M*-value of 0.015; *PP2A* and *EF-1a* were the least stable genes. Generally, *SAND* and *PGK* were the most stable genes in all experimental conditions and could be used as housekeeping genes.

The pairwise variation (*V*) was also calculated to determine the optimal number of genes required for normalization. The V_n_/V_n__+1_-values were far <0.15 under all experimental conditions, indicating that one stable reference gene is enough to obtain accurate results.

### NormFinder

The NormFinder program is used to calculate an arbitrary stability value and standard error, considering the intragroup and intergroup variations of each reference gene in all groups (Andersen et al., 2004). The stability values of reference genes were calculated by NormFinder, as shown in Table 5. The ranking order generated by this method was somewhat similar to the results determined by GeNorm. *SAND* and *PGK* were still the two most stable genes among all tested samples.

**Table 5:** **Ranking of reference genes and their expression stability values calculated using NormFinder**.

**Rank**	**Total**		**set1**		**set2**		**set3**	
	**Gene**	**Stability**	**Gene**	**Stability**	**Gene**	**Stability**	**Gene**	**Stability**
1	*SAND*	0.011	*SAND*	0.007	*SAND*	0.009	*PGK*	0.005
2	*PGK*	0.012	*PGK*	0.013	*PGK*	0.009	*F-box*	0.009
3	*F-box*	0.015	*TUB*	0.013	*F-box*	0.010	*SAND*	0.012
4	*Actin*	0.022	*Actin*	0.019	*EF-1a*	0.012	*Actin*	0.021
5	*UBQ*	0.022	*EF-1a*	0.021	*MTP*	0.015	*UBQ*	0.022
6	*PP2A*	0.023	*UBQ*	0.023	*PP2A*	0.019	*MTP*	0.023
7	*TUB*	0.030	*F-box*	0.023	*Actin*	0.021	*TUB*	0.028
8	*EF-1a*	0.034	*PP2A*	0.025	*UBQ*	0.021	*PP2A*	0.029
9	*MTP*	0.036	MTP	0.028	TUB	0.030	*EF-1a*	0.029

### Bestkeeper

Bestkeeper ranks the reference genes according to the standard deviation (*SD*) of their Cqs, and the output includes more information, for example, the coefficient of variation (*CV*) and correlation (*r*) (Pfaffl et al., 2004). If the *SD*-values exceed 1.0, reference genes are considered to be unstable and should be avoided for gene expression normalization. Our results indicated that most genes had *SD*-values smaller than 1.0, including eight genes in the total, nine genes in set1, nine genes in set2, and three genes in set3 (Table 6). The ranking order was quite different from the results calculated by GeNorm and NormFinder, but the *SD*-values of *PKG* and *SAND* were smaller than one in all sets.

**Table 6:** **Ranking of reference genes and their ***SD*** calculated using Bestkeeper**.

**Rank**	**Total**		**set1**		**set2**		**set3**	
	**Gene**	***SD***	**Gene**	***SD***	**Gene**	***SD***	**Gene**	***SD***
1	*PP2A*	0.73	*PP2A*	0.35	*PP2A*	0.73	*UBQ*	0.86
2	*UBQ*	0.78	*UBQ*	0.42	*EF-1a*	0.74	*Actin*	0.87
3	*SAND*	0.83	*TUB*	0.43	*TUB*	0.84	*SAND*	0.93
4	*TUB*	0.88	*Actin*	0.47	*UBQ*	0.87	*PGK*	1
5	*Actin*	0.9	*MTP*	0.51	*MTP*	0.9	*MTP*	1.07
6	*EF-1a*	0.92	*SAND*	0.54	*PGK*	0.93	*EF-1a*	1.09
7	*PGK*	0.93	*EF-1a*	0.55	*Actin*	0.94	*F-box*	1.13
8	*MTP*	0.94	*PGK*	0.57	*F-box*	0.97	*TUB*	1.17
9	*F-box*	1.02	*F-box*	0.85	*SAND*	0.99	*PP2A*	1.28

### Ref-finder

Finally, Ref-finder was used to generate an integrated ranking of the most stable candidate reference genes (Table 7). Differences of results were found among methods and slight disparities were also found when analysis with single method compared with result of integration. However, the most stable gene was roughly identical. *SAND* was found to be the most stably expressed gene in all samples and in set1. Both *SAND* and *PGK* were the most stable in set2, and *PGK* was the most stable in set3. *MTP* was the least stable gene in all samples and in set1. *Actin* and *PP2A* was the least stable gene in set2 and set3, respectively.

**Table 7:** **Stability ranking of nine candidate reference genes**.

**Methods**	**1**	**2**	**3**	**4**	**5**	**6**	**7**	**8**	**9**
**TOTAL**									
Recommended comprehensive ranking	*SAND*	*PGK*	*UBQ*	*PP2A*	*F-box*	*Actin*	*TUB*	*EF-1a*	*MTP*
GeNorm	*SAND*/ *PGK*		*F-box*	*UBQ*	*Actin*	*PP2A*	*TUB*	*EF-1a*	*MTP*
NormFinder	*SAND*	*PGK*	*UBQ*	*F-box*	*PP2A*	*Actin*	*TUB*	*EF-1a*	*MTP*
Bestkeeper	*PP2A*	*UBQ*	*SAND*	*TUB*	*Actin*	*EF-1a*	*PGK*	*MTP*	*F-box*
**SET1**									
Recommended comprehensive ranking	*SAND*	*TUB*	*PGK*	*EF-1a*	*UBQ*	*PP2A*	*Actin*	*F-box*	*MTP*
GeNorm	*EF-1a/ PGK*		*SAND*	*TUB*	*F-box*	*Actin*	*UBQ*	*PP2A*	*MTP*
NormFinder	*SAND*	*TUB*	*PGK*	*EF-1a*	*Actin*	*UBQ*	*F-box*	*PP2A*	*MTP*
Bestkeeper	*Actin*	*EF-1a*	*MTP*	*PP2A*	*SAND*	*TUB*	*UBQ*	*PGK*	*F-box*
**SET2**									
Recommended comprehensive ranking	*UBQ*	*PGK*	*SAND*	*EF-1a*	*PP2A*	*MTP*	*F-box*	*TUB*	*Actin*
GeNorm	*SAND/PGK*		*MTP*	*UBQ*	*F-box*	*EF-1a*	*Actin*	*PP2A*	*TUB*
NormFinder	*UBQ*	*PGK*	*SAND*	*F-box*	*EF-1a*	*MTP*	*PP2A*	*Actin*	*TUB*
Bestkeeper	*Actin*	*EF-1a*	*MTP*	*PP2A*	*SAND*	*TUB*	*UBQ*	*F-box*	*PGK*
**SET3**									
Recommended comprehensive ranking	*PGK*	*SAND*	*UBQ*	*F-box*	*Actin*	*MTP*	*EF-1a*	*TUB*	*PP2A*
GeNorm	*SAND*/*PGK*		*F-box*	*UBQ*	*MTP*	*Actin*	*EF-1a*	*TUB*	*PP2A*
NormFinder	*PGK*	*F-box*	*SAND*	*UBQ*	*Actin*	*MTP*	*EF-1a*	*TUB*	*PP2A*
Bestkeeper	*Actin*	*SAND*	*MTP*	*PP2A*	*TUB*	*EF-1a*	*UBQ*	*F-box*	*PGK*

### Reference gene in varieties

Furthermore, we analyzed the stability in varieties by four algorithms (Bestkeeper, NormFinder, GeNorm, and Ref-finder). There is difference among varieties. *PGK* was the most stable reference gene in LFC2, LFC3, PC1, and PC24, and thus *PGK* could be a good candidate gene in chrysanthemums. *F-box, Actin*, and *MTP* performed well in LFC1, LFC4, and PC3, respectively. Still, *PGK* and *SAND* rank higher in most varieties (Table 8).

**Table 8:** **Stability ranking of nine candidate reference genes in varieties of chrysanthemum**.

**Rank**	**LFC1**	**LFC2**	**LFC3**	**LFC4**	**PC1**	**PC2**	**PC3**
1	*F-box*	*PGK*	*PGK*	*Actin*	*PGK*	*PGK*	*MTP*
2	*PGK*	*UBQ*	*UBQ*	*SAND*	*SAND*	*F-box*	*SAND*
3	*SAND*	*F-box*	*EF-1a*	*F-box*	*EF-1a*	*Actin*	*F-box*
4	*EF-1a*	*SAND*	*F-box*	*EF-1a*	*F-box*	*UBQ*	*PGK*
5	*UBQ*	*EF-1a*	*SAND*	*PP2A*	*UBQ*	*EF-1a*	*EF-1a*
6	*MTP*	*PP2A*	*MTP*	*UBQ*	*Actin*	*SAND*	*UBQ*
7	*TUB*	*MTP*	*PP2A*	*PGK*	*MTP*	*TUB*	*TUB*
8	*PP2A*	*TUB*	*Actin*	*MTP*	*PP2A*	*MTP*	*PP2A*
9	*Actin*	*Actin*	*TUB*	*TUB*	*TUB*	*PP2A*	*Actin*

**Table 9:** **Sequences of ***DmDFR*** and ***LCYE*** for RT-PCR**.

**Name**	**Forward primer sequences(5′–3′)**	**Reverse primer sequences(5′–3′)**	**Amplicon length(bp)**	**Tm (°C)**
*LCYE*	GGAGCGGCTTCGGGTAAACTTCTGCAA	CTCTCTTGAAGCCAGACAGGTTTCCTC	260	60
*DmDFR*	TTGGCGGAGAAAGCAGCA	AGACTTGGTGGGAACGAGGG	115	56.2

### Reference gene validation

**Figure 4:**
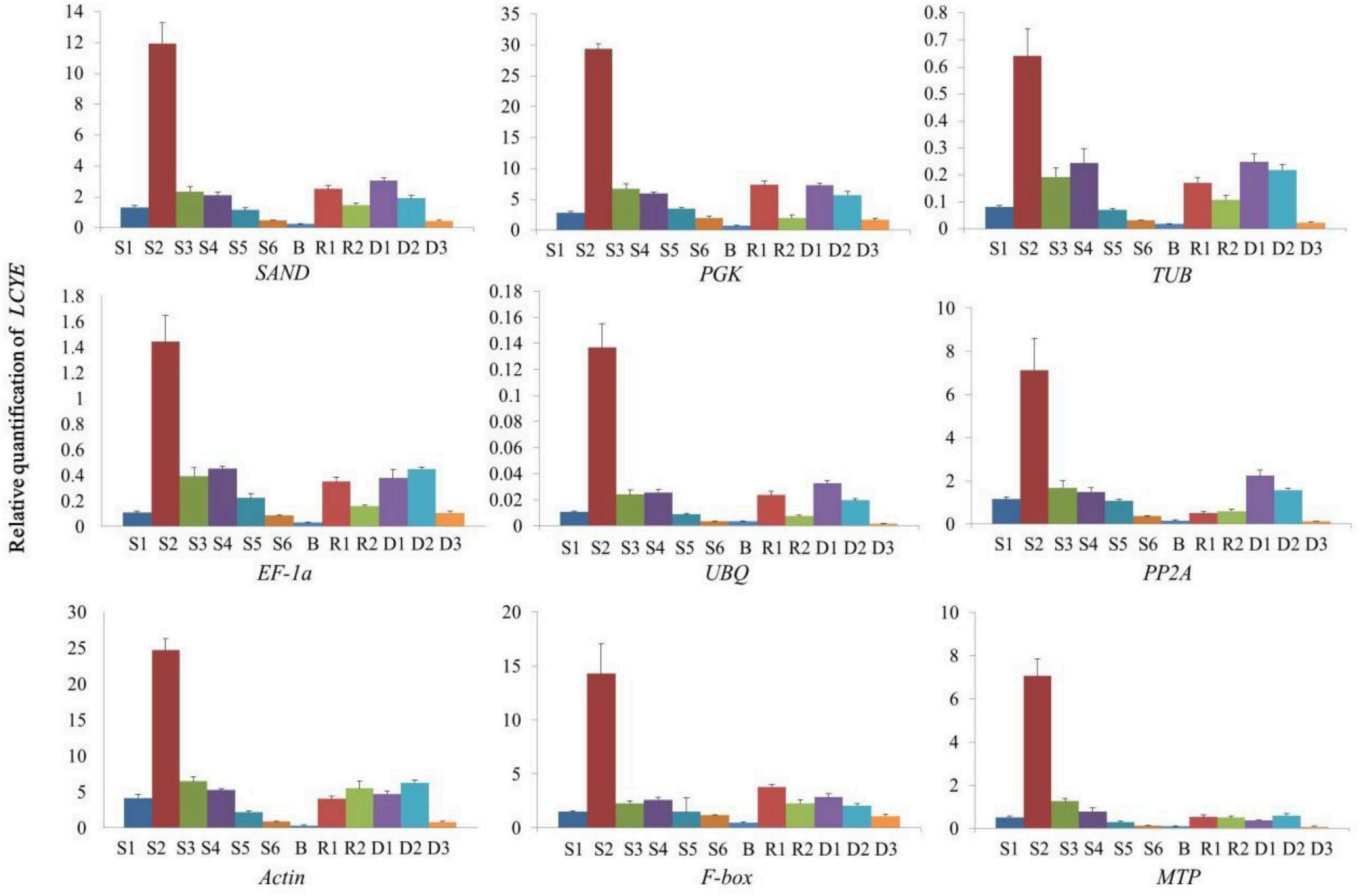
**Relative quantification of ***LCYE*** expression in different tissues of ***C. lavandulifolium*** with competence to flower using validated reference genes for normalization**.

Firstly, we quantified the expression of *LCYE* in *C. lavandulifolium* using the selected reference genes. When normalized using the two most stable genes (*SAND* and *PGK*) as internal controls, the relative expression levels were the same. The result showed that in *C. lavandulifolium*, the relative expression level was significantly increased in S2 and then decreased with flower development. The same pattern was shown in the ray floret and disc floret, and the relative expression level was lower in green bracts. When normalized using *TUB*, which was the third most stable gene as calculated, the expression pattern was slightly changed. The relative expression level in S4 was higher than that in S3, which is contradictory to the above patterns. The expression pattern of genes latter in the ranking order show more differences (Figure 4).

**Figure 5:**
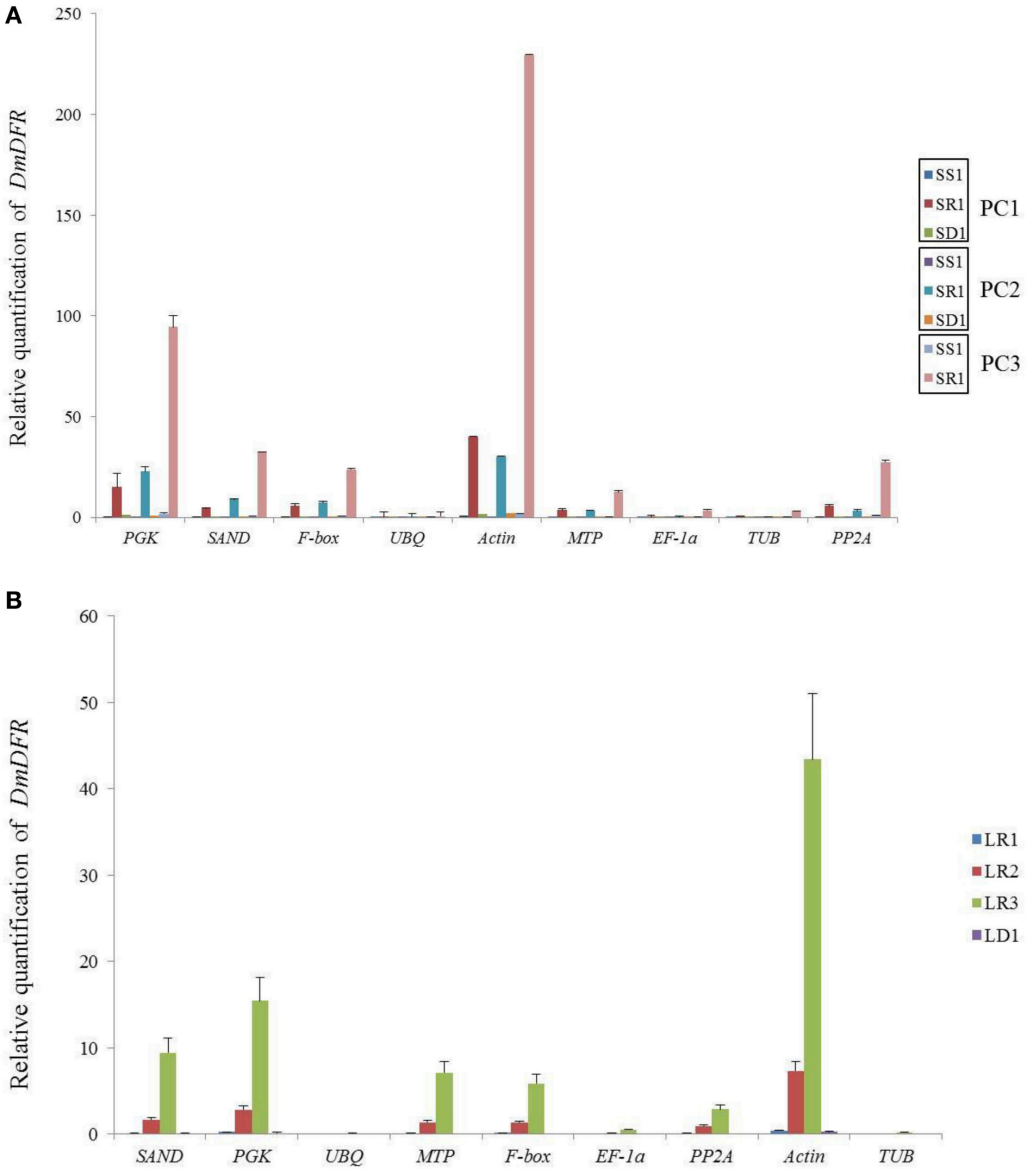
**Relative mRNA levels of ***DmDFR*** in different organs of red chrysanthemums. (A)** Relative mRNA levels of *DmDFR* in set3, **(B)** Relative mRNA levels of *DmDFR* in LC1.

**Figure 6:**
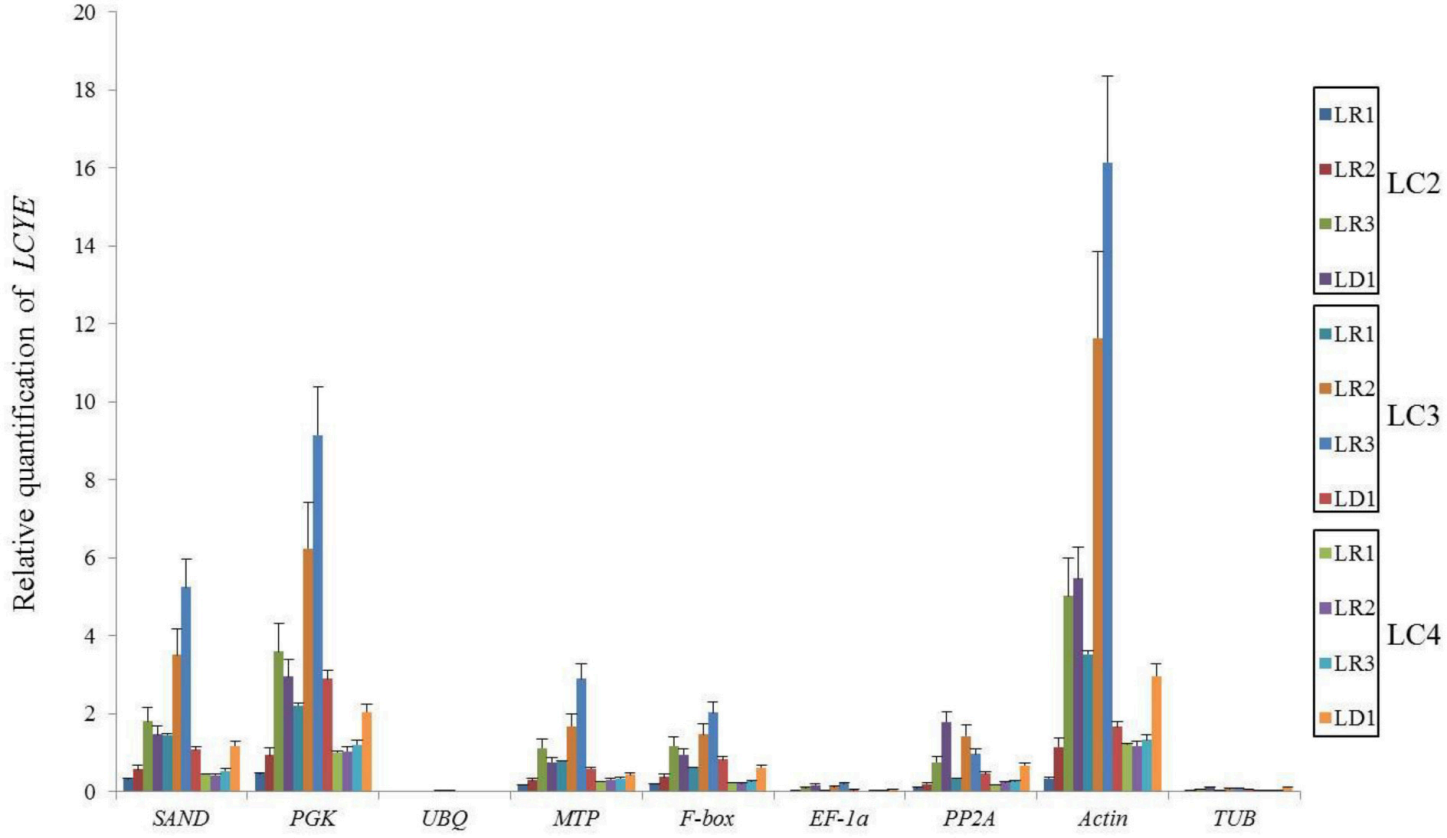
**Relative mRNA levels of ***LCYE*** in different organs of yellow chrysanthemums**.

We chose the most stable gene to normalize the expression level in different cultivars. The expression pattern of *DmDFR* was correlated with the color. It was more highly expressed in the red petals of ray florets, and there was almost no expression in disc florets and flower buds (Figure 5A). *DmDFR* expression increased during petal development in LFC1 (Figure 5B). *LCYE* was expressed in both ray florets and disc florets of yellow chrysanthemums and exhibited some differences among varieties. *LCYE* was more highly expressed in ray florets than disc florets, and its expression increased during petal development in LFC1 and LFC2. Nevertheless, it was more highly expressed in disc florets than ray florets and was expressed steadily during petal development in LFC3, which is typical of Anemone (Figure 6). By the results, it showed that there were some divergence among results calculated by different reference.

## Discussion

qRT-PCR has become an important technique for the analysis of gene expression because of its sensitivity, accuracy, and high throughput nature (Bustin, 2000). It is necessary to select the proper candidate reference genes as there are no universally-suitable reference genes. The ideal reference genes should be stably expressed under all experimental conditions and independent of organs, tissues, developmental stages, and different treatments (Huggett et al., 2005).

In this study, the stability of nine reference genes in various tissues during flower development in *C. morifolium* and C. *lavandulifolium* was analyzed. The structure of the capitulum in chrysanthemums is very special. The capitula of chrysanthemums consist of ray florets and disc florets, which are quite different in shape and gender. It is a great material for flower development research in higher plants. Several reference gene validation attempts have been reported for Asteraceae (Gu et al., 2011; Fu et al., 2013; Hong and Dai, 2015; Wang et al., 2015). They mainly focus on flower color, flowering and cross-species. There is no detailed validation has been conducted to test their stability in different organs during flower development. We selected nine genes as candidate reference genes, including genes involved in protein degradation (*MTP*), the regulation of phosphorylation (*PP2A*), determining cytoskeletal structure (*TUB* and *ACTIN*), protein synthesis (*EF-1a*) and others, including genes encoding a protein kinase (*PGK*), ubiquitin (*UBQ*), SAND family protein (*SAND*), and F-box family protein (*F-box*). Some of them are traditional reference genes and the others are new reference genes. A great deal of experiments in *A. thaliana* (Czechowski et al., 2005), *Coffea Arabica* (Cruz et al., 2009), *Gossypium hirsutum* (Artico et al., 2010) proved that the new reference genes were better for use than the conventional genes.

A good RT-qPCR experiment should always be based on a well-thought sampling protocol (De Keyser et al., 2013). Rieu and Powers (2009) proposed that, in ideal condition, all of the reactions should be run on the same plate. In addition, randomized block design is a better strategy considering the accuracy of statistic. In this work, we performed the program for each gene in a single plate, while, three independent biological replicates were conducted to diminish the error. The samples were collected on different flowers of at least three plants and RNA were extracted at the same time with same methods. Rieu and Powers further pointed out that the current generation of real-time PCR cyclers had a better performance in reducing the within-plate variation introduced during the PCR run. As a result, nonrandom plate setup could be a more practical method.

There is no consensus as to which type of algorithm should be used to determine the stability of gene expression because different statistical methods based on different principles yield potentially contradictory results from the same data. Therefore it is important to use at least three different softwares in order to achieve best results as possible in particular to avoid selection of co-regulated genes (Jacob et al., 2013). In this study, we used three frequently used statistical algorithms, GeNorm, NormFinder, and Bestkeeper, to analyze the stability of candidate reference genes. The optimal reference genes were determined by synthesizing using the above described methods. GeNorm and NormFinder are the most widely used algorithms, and they were different from each other in many studies, such those in as flax (Huis et al., 2010), rubber tree, tobacco (Schmidt and Delaney, 2010), and Oxytropis ochrocephala Bunge (Zhuang et al., 2015). However, there are still some studies in which these algorithms showed very few differences from each other, such as those in coffee (Cruz et al., 2009) and rice (Jain, 2009). There were reports that both NormFinder and GeNorm have minor differences in gene stability ranking (Jain et al., 2006; Cruz et al., 2009). NormFinder was more effective than GeNorm in wheat (Paolacci et al., 2009). The results of GeNorm and NormFinder were similar, but they showed some differences from the results obtained with Bestkeeper. *SAND* and *PGK* are the most stable genes when calculated by GeNorm and NormFinder. Additionally, their *SD*s were <1 when calculated by Bestkeeper. Thus, *SAND* and *PGK* are stable and available in our samples. Finally, we used Ref-finder to combine and validate the results. There are some differences among algorithms, but they are not obvious.

The results highlight the fact that it is better to select different reference genes depending on the biological samples. In conclusion, based on the results of the present study, *SAND* and *PGK* were good candidates for normalization in all samples, *C. lavandulifolium* and Chinese large-flowered chrysanthemum cultivars. The results of this study suggest that *PGK* yielded low *M*-values in different tissues during the developmental process of potted chrysanthemums. Functional studies suggest, that the SAND family proteins are involved in late steps of endocytic transport (Cottage et al., 2004; Poteryaev and Spang, 2005). *SAND* was revealed as one of the superior reference genes found for proper normalization in a large number of studies, such as in tomato development studies (Expósito-Rodríguez et al., 2008), in a set of organs and tissues of buckwheat (Demidenko et al., 2011), in different citrus organs and following different biotic stresses (Mafra et al., 2012), in *Petunia hybrida* during leaf and flower development (Mallona et al., 2010), in tissues during berry development (Ye et al., 2015) and in abiotic stress samples of *Syntrichia caninervis* (Li et al., 2015). *PGK* plays important roles in the glycolytic pathway (Anderson and Carol, 2005). *PGK* (phosphoglycerate kinase) was one of the superior reference genes found for proper normalization in chrysanthemum of cross-ploidy levels (Wang et al., 2015). *MTP* and *EF-1a* yielded poor values in all sample sets in this work, although its homolog in soybeans is one of the most optimal genes in different tissues and photoperiod treatment samples (Jian et al., 2008; Hu et al., 2009). There were differences for best reference genes among varieties as the genetic background of varieties were complex. *PGK* and *SAND* yielded low *M*-values in most varieties, although there are exceptions, such as *F-box, Actin*, and *MTP*, which was most stable in LFC1, LFC4, and PC3, respectively. It is advised to select reference genes for specific varieties. However, in general, *PGK* and *SAND* were the best genes among all samples, not only in varieties of different petal types but also in stages and tissues of flower development.

We quantified the expression of *DmDFR* and *LCYE* genes. *DmDFR* and *LCYE* play important roles in the anthocyanin biosynthesis pathway and carotenoid biosynthesis pathway, respectively (Kishimoto and Ohmiya, 2006; Huang et al., 2013). *LCYE* showed increasing levels of expression during petal development in the petals of the yellow chrysanthemum, and its pattern was well correlated with the pattern of carotenoid accumulation (Kishimoto and Ohmiya, 2006). Molecular breeding of *DFR* has been used to create blue colored flowers in roses and carnations (Katsumoto et al., 2007; Tanaka et al., 2009). The results showed that the expression pattern of *DmDFR* and *LCYE* were well correlated with the color. Interestingly, we found that there were differences in the expression pattern in ray florets and disc florets among varieties. For example, *LCYE* was more highly expressed in ray florets than in disc florets, and its expression increased during petal development in LFC2 and LFC3. Nevertheless, it was more highly expressed in disc florets than in ray florets and was expressed steadily during petal development in LFC4. They exhibit the same color with different pigment distribution.

This research analyzed reference genes of flower development in *C. morifolium* and *C. lavandulifolium* for RT-qPCR and discussed the expression of genes involved in flower color. It will provide basic data for molecular research of flower development in chrysanthemums.

## Author contributions

Designed and performed and the experiments: SQ, SD; Analyzed data and wrote the paper: SQ, LY, XW, and YH; Conducted the experiments: SQ; Materials support: XS, MZ.

### Conflict of interest statement

The authors declare that the research was conducted in the absence of any commercial or financial relationships that could be construed as a potential conflict of interest.

## Supplementary material

Table S1 **The Ct value of primers**.

Figure S1 **The melt peak of primers**.

Figure S2 **The melt curve of primers**.

Figure S3 **The ten-fold serial dilution curves of primers**.
