# Supplementary figures and images for: Reference Gene Selection for RT-qPCR Analysis of Flower Development in Chrysanthemum morifolium and Chrysanthemum lavandulifolium
Source: Front Plant Sci. 2016 Mar 11;7:287. doi: 10.3389/fpls.2016.00287 (PMC4786574; doi:10.3389/fpls.2016.00287)

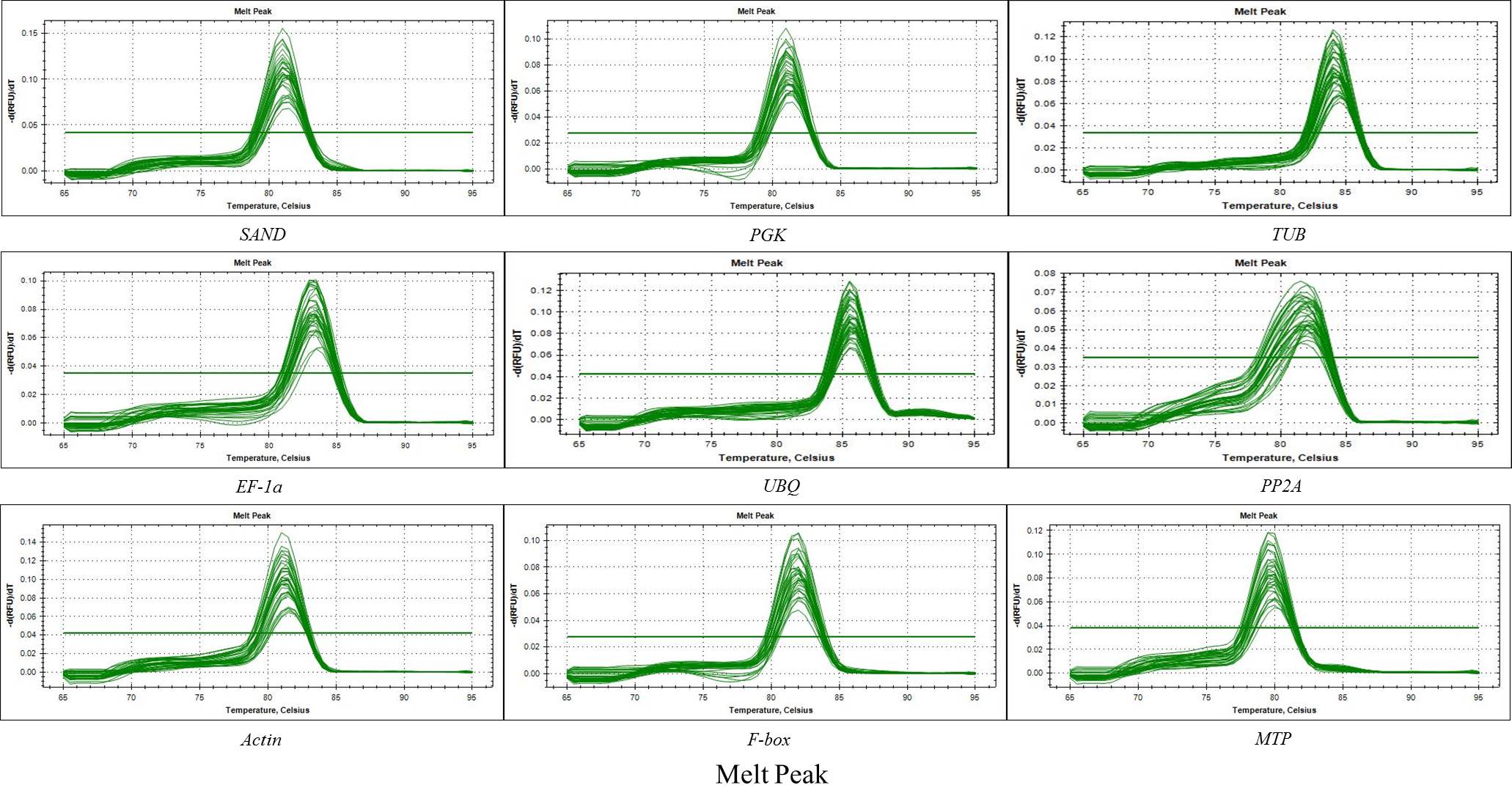

Supplement: Figure S1 — The melt peak of primers. [file Image1.JPEG]

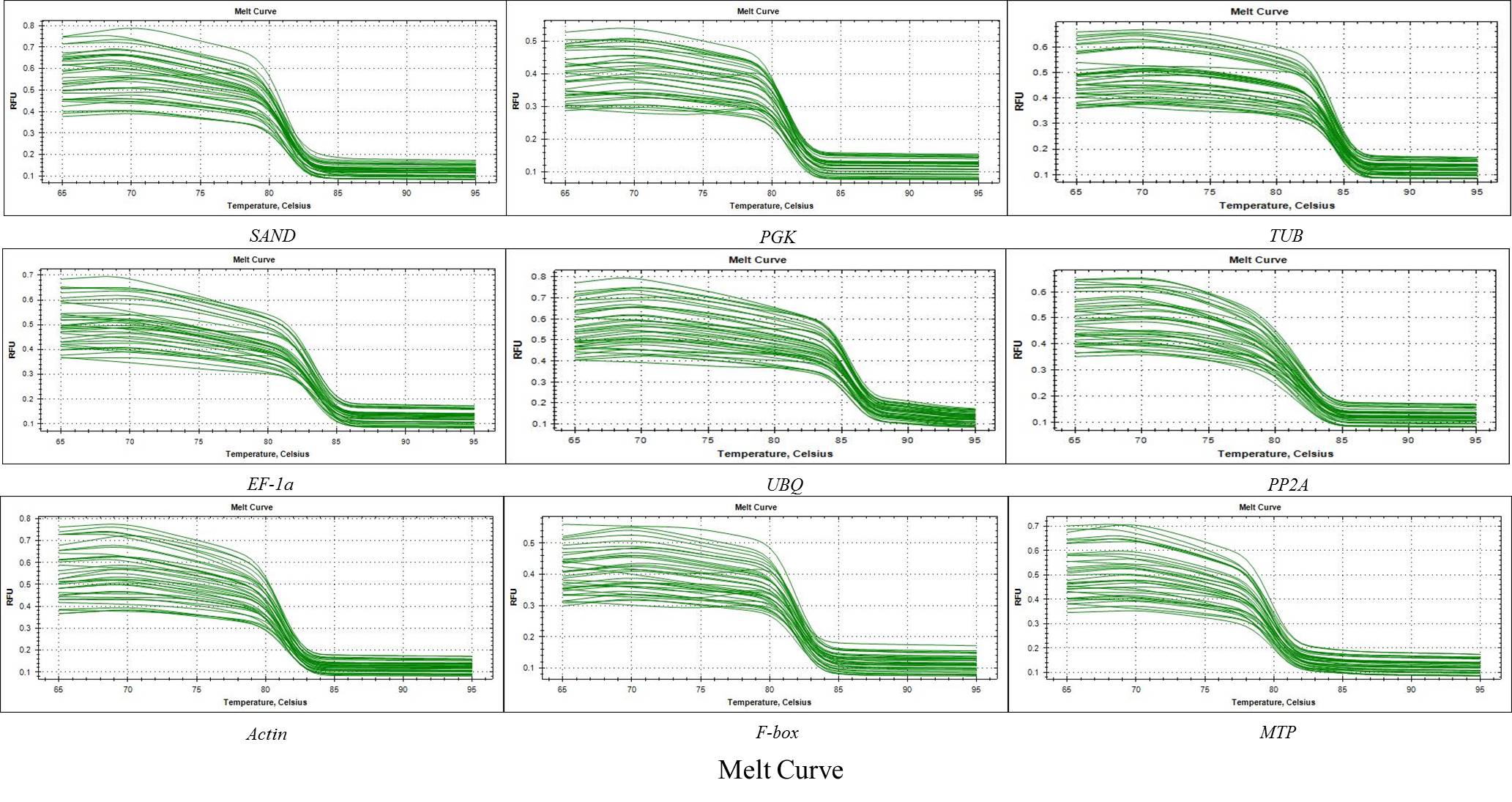

Supplement: Figure S2 — The melt curve of primers. [file Image2.JPEG]

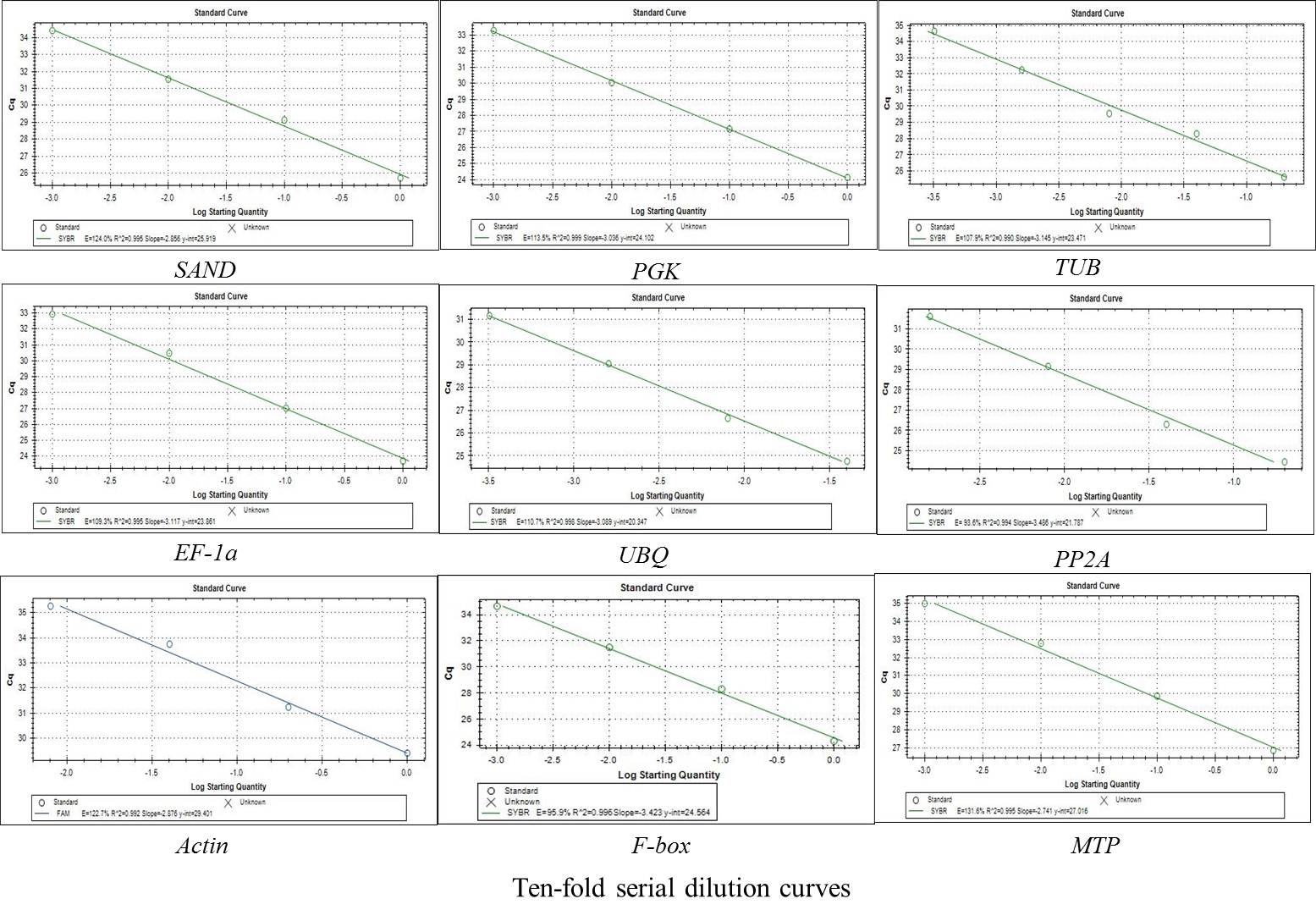

Supplement: Figure S3 — The ten-fold serial dilution curves of primers. [file Image3.JPEG]
